# Alterations in leukocyte DNA methylome are associated to immunosuppression in severe clinical phenotypes of septic patients

**DOI:** 10.3389/fimmu.2023.1333705

**Published:** 2024-01-03

**Authors:** Jesús Beltrán-García, Germán Casabó-Vallés, Rebeca Osca-Verdegal, Paula Navarrete-López, María Rodriguez-Gimillo, Elena Nacher-Sendra, Carolina Ferrando-Sánchez, Eva García-López, Federico V. Pallardó, Nieves Carbonell, Salvador Mena-Mollá, José Luis García-Giménez

**Affiliations:** ^1^Center for Biomedical Network Research on Rare Diseases (CIBERER), Carlos III Health Institute, Valencia, Spain; ^2^INCLIVA Biomedical Research Institute, Valencia, Spain; ^3^Department of Physiology, Faculty of Medicine and Dentistry, University of Valencia, Valencia, Spain; ^4^Department of Medicine, University of California, San Diego, San Diego, CA, United States; ^5^EpiDisease S. L. (Spin-Off CIBER-ISCIII), Parc Científic de la Universitat de València, Valencia, Spain; ^6^Salk Institute for Biological Studies, San Diego, CA, United States; ^7^Department of Animal Reproduction, Instituto Nacional de Investigación y Tecnología Agraria y Alimentaria (INIA)-Centro Superior de Investigaciones Científicas (CSIC), Madrid, Spain; ^8^Intensive Care Unit, Hospital Clínico Universitario de Valencia (HCUV), Valencia, Spain

**Keywords:** DNA methylation, immunosuppression, inflammation, immune system, sepsis

## Abstract

**Introduction:**

Sepsis patients experience a complex interplay of host pro- and anti-inflammatory processes which compromise the clinical outcome. Despite considering the latest clinical and scientific research, our comprehension of the immunosuppressive events in septic episodes remains incomplete. Additionally, a lack of data exists regarding the role of epigenetics in modulating immunosuppression, subsequently impacting patient survival.

**Methods:**

To advance the current understanding of the mechanisms underlying immunosuppression, in this study we explored the dynamics of DNA methylation using the Infinium Methylation EPIC v1.0 BeadChip Kit in leukocytes from patients suffering from sepsis, septic shock, and critically ill patients as controls, within the first 24 h after admission in the Intensive Care Unit of a tertiary hospital.

**Results and discussion:**

Employing two distinct analysis approaches (DMRcate and mCSEA) in comparing septic shock and critically ill patients, we identified 1,256 differentially methylated regions (DMRs) intricately linked to critical immune system pathways. The examination of the top 100 differentially methylated positions (DMPs) between septic shock and critically ill patients facilitated a clear demarcation among the three patient groups. Notably, the top 6,657 DMPs exhibited associations with organ dysfunction and lactate levels. Among the individual genes displaying significant differential methylation, IL10, TREM1, IL1B, and TNFAIP8 emerged with the most pronounced methylation alterations across the diverse patient groups when subjected to DNA bisulfite pyrosequencing analysis. These findings underscore the dynamic nature of DNA methylation profiles, highlighting the most pronounced alterations in patients with septic shock, and revealing their close association with the disease.

## Introduction

Sepsis is a critical condition that occurs when the exacerbated body’s response against an infective microorganism damages its own tissues, leading to organ dysfunction and increased risk of mortality (1). The response of the immune system during sepsis is a complex, dynamic, time-dependent process involving simultaneous hyper-inflammatory and anti-inflammatory responses. The exacerbated hyper-inflammatory response can also damage non-infected tissues and lead to dysfunction of different organs and systems linked to hypotension, vasculature damage, and coagulation events, aggravating the disease status of septic patients, and producing early deaths at the initial stages of sepsis (2). The hyperinflammatory process is an acute event occurring usually at initial stages of sepsis just after infection (3–5). A compensatory anti-inflammatory response syndrome (CARS) can sometimes simultaneously occur, which can induce long-term immunosuppression, leading to secondary infections and most sepsis-associated late deaths (6, 7).

Immunosuppression has previously been linked to extensive leukocyte apoptosis during simultaneous hyper-inflammatory and anti-inflammatory responses [e.g., bone marrow (8), thymus (9), spleen (10, 11) and, lymphoid tissues]. Other mechanisms such as production of anti-inflammatory mediators like interleukin (IL)-10 (12) and T_H_1-opposing T_H_2 cytokines like IL-4 (13) are also heightened during the anti-inflammatory response (14), contributing to immunosuppression. Regulatory T (Treg) cells, central players in maintaining immunological homeostasis and tolerance, have also been proposed as contributing to sepsis-induced immunosuppression. Indeed, circulating Treg cell percentages are markedly increased in septic patients (15). Likewise, Myeloid-derived suppressor cells (MDSCs) are also involved in sepsis-induced immunosuppression throughout the production of arginase 1 and contribute to T-cell dysfunction (16). Importantly, levels of MDSCs mediators and the expression of genes with immunosuppressive functions, such as *S100A8/A9*, *S100A12*, and *ARG1*, are highly elevated in patients with sepsis (3, 16). Despite accumulating scientific evidence on the pathogenetic mechanisms underlying sepsis, data explaining sepsis-induced immunosuppression is still lacking, and the molecular mechanisms occurring in the host leading to immunosuppression in human sepsis remain poorly understood (6, 17, 18).

The profound impact of epigenetics on immune system development and regulation, coupled with its intricate connection to disease severity through inflammatory phenotype regulation, highlights the importance of investigating epigenetic mechanisms to understand the pathophysiology of sepsis events and immunosuppression (19, 20). In this regard, as an epigenetic mechanism, DNA methylation holds a prominent position in the extensive body of research on inflammation, immunomodulation, immune cell differentiation involving lineage-specific gene repression or activation, as well as immunosuppression processes (21).

In this study, we explore the impact of DNA methylation on altered biological processes during sepsis and septic shock. By conducting a high-throughput comprehensive analysis of DNA methylation sites and regions across the genome, we identify key genes included in critical pathways associated with the immunosuppression observed in septic patients. Our findings provide valuable insights into the mechanisms underlying sepsis-induced immunosuppression, potentially laying the groundwork for novel therapeutic strategies aimed at addressing this life-threatening condition.

## Materials and methods

### Human samples

We selected patients with sepsis and septic shock following the Third International Consensus Definitions for Sepsis and Septic Shock (Sepsis-3) (1). In each patient, we analyzed different clinical parameters and calculated the Sequential [Sepsis-related] Organ Failure Assessment (SOFA) score.

Sepsis and septic shock patients were recruited from the Intensive Care Unit (ICU) of the *Hospital Clínico Universitario de Valencia* (Valencia, Spain), to which we added a control group with non-infectious systemic inflammatory response syndrome (SIRS), specifically critically ill ICU patients with spontaneous intracerebral cerebral hemorrhage (ICH). First, we used a discovery cohort (n=12) composed of five septic shock, three septic, and four critically ill patients (CIP). Afterwards, we used a validation cohort (n=30) composed of eleven septic shock patients, nine septic patients, and nine CIP. Study patients’ clinical data are summarized in **Table 1** (discovery cohort) and **Table 2** (validation cohort). Blood samples were collected during the first 24 h of sepsis diagnosis and processed in the INCLIVA biobank.

**Table 1:** Clinical data from critically ill (CIP), sepsis, and septic shock patients in the discovery cohort.

Participant characteristics	CIP (n =4)	Sepsis (n = 3)	Septic shock (n = 5)	p-value
Age (years) Median (IQR)	65 (34)	61 (12)	62 (7)	n.s
APACHE II score Median (IQR)	6 (7)	17 (2)	16 (5)	n.s
SOFA score 1^st day^ Median (IQR)	5 (2.5)	4.0 (6.0)	8.0 (5.5)	0.020*
CRP (mg/l) median (IQR)	4.8 (14.8)	431.0 (541.0)	160.0 (339.5)	0.022^#^
Procalcitonin (ng/mL) median (IQR)	–	2.1 (23.0)	20.4 (72.0)	n.s
Lactate 1^st hour^ (mmol/l) median (IQR)	1.1 (1.6)	1.7 (0.9)	3.4 (5.5)	0.016^&^
Number of patients with antimicrobial therapy in the first 6h	0	3	5	–
Lactate 6h (mmol/l) median (IQR)	–	1.3 (0.5)	2 (2.7)	n.s
ICU mortality n (%)	0 (0)	0 (0)	0 (0)	n.s.

Spearman’s rho test was used to perform the different statistical analyses between clinical variables. IQR, interquartile range. N.s, no statistically significant differences. *Comparison septic shock vs. CIP; ^#^Comparison sepsis vs. CIP; ^&^Comparison septic shock vs sepsis. CIP, critically ill patients with non-related sepsis disease.

APACHE, Acute Physiology and Chronic Health Evaluation; SOFA, Sequential Organ Failure Assessment; CRP, C-reactive protein.

**Table 2:** Detailed clinical data from critically ill (CIP), sepsis, and septic shock patients in the validation cohort.

Participant characteristics	CIP (n = 9)	Sepsis (n = 9)	Septic shock (n = 11)	p-value
Age (years) Median (IQR)	65 (14)	72 (10)	70 (14)	n.s
APACHE II score Median (IQR)	15 (8)	14 (5)	26 (5)	0.0030* 0.0030^#^
SOFA score 1^st day^ Median (IQR)	3.0 (5.0)	5.0 (5.0)	10.0 (5.0)	0.0006* 0.0006^#^
CRP (mg/l) median (IQR)	4.8 (7.4)	270.0 (247.0)	235.0 (179.0)	0.0003* 0.0003^#^
Procalcitonin (ng/mL) median (IQR)	–	2.1 (6.2)	36.5 (59.0)	0.0016^&^
Lactate 1^st hour^ (mmol/l) median (IQR)	1.8 (0.6)	1.4 (0.5)	8.0 (1.5)	0.0090^&^
Lactate 6h (mmol/l) median (IQR)	–	1.6 (0.5)	1.5 (2.1)	n.s
Number of patients with antimicrobial therapy in the first 6h	0	9	11	–
ICU mortality n (%)	2 (22)	2 (22)	5 (45.5)	n.s

Spearman`s rho test was used to perform the different statistical analyses between clinical variables. IQR, interquartile range. N.s., no statistically significant differences *Comparison septic shock vs. CIP; ^#^Comparison sepsis vs. CIP; ^&^Comparison septic shock vs. sepsis. CIP, critically ill patients with non-related sepsis disease.

### DNA purification and methylation analysis using EPIC 850K methylation arrays

DNA was purified from leukocytes of patients’ whole blood collected in anti-coagulant (EDTA K2) tubes. All samples were collected within the first 24 hours of ICU admission. DNA was extracted using DNeasy Blood & Tissue Kit (Qiagen Inc., Germantown, MD USA).

One μg of genomic DNA was treated with bisulfite conversion using the EZ-96 DNA Methylation Kit (Zymo Research, Irvine, CA, USA) following the manufacturer’s recommendations for Infinium assays. Afterwards, DNA methylation analysis was performed with the Infinium MethylationEPIC v1.0 BeadChip (Illumina Inc, San Diego, CA, USA), used to analyze >850000 CpG sites, which covers 99% of genes described and 95% of CpG islands, including data from projects such as ENCODE and FANTOM5.

In brief, 4 µL of bisulfite-converted DNA were used following the Illumina Infinium HD Methylation Assay protocol as previously described (22). First, we performed a whole genome amplification step followed by enzymatic endpoint fragmentation, precipitation, and resuspension. Afterwards, processed samples were hybridized on Infinium MethylationEPIC v1.0 BeadChips at 48°C for 16 h. Unhybridized and non-specifically hybridized DNA were washed away. To this chemical reaction we next added a single nucleotide extension, using the hybridized bisulfite-treated DNA as a template, nucleotides labeled with biotin (ddCTP and ddGTP), and 2,4-dinitrophenol (ddATP and ddTTP). After the single base extension, several repeated rounds of staining were performed with a combination of antibodies that differentiated DNP and biotin by fixing them with different fluorophores. Finally, the BeadChip was washed and protected for scanning on the Illumina HiScan SQ scanner (Illumina Inc, San Diego, CA, USA).

### Bioinformatic analysis

The minfi R-package (23) was used to read raw IDAT files obtained from the Illumina EPIC array, assess their quality, and perform the normalization and the exclusion of probes that might interfere in subsequent analysis (24). DNA methylation probes were filtered to remove those with poor detection p-values (< 0.01) in any sample, those that matched with previously described specific SNP positions, sex-related probes (X & Y chromosome) and those described as multiple reactive probes (25).

After that, a differential methylation analysis was performed to identify both differentially methylated positions (DMPs) and regions (DMRs) between the 3 groups of patients. We compared septic shock patients vs. non-septic critically ill patients (CIP), sepsis patients vs. CIP, and septic shock patients vs. sepsis patients.

The position level methylation analysis was carried out using the limma R-package (26). To adjust for multiples comparisons, CpG probes with a False Discovery Rate (FDR) < 0.05 were considered significant. Subsequently, the methylation analysis was performed at region level using two different approaches. On the one hand, we used the methylated CpGs Set Enrichment Analysis (mCSEA) R-package (27), which implements a method based on the Gene Set Enrichment Analysis (GSEA) to identify subtle but consistent differentially methylated regions (DMRs) in complex phenotypes among different groups such as those in this work (critically ill, sepsis, and septic shock patients). DMRs are associated to genes and to promoters, and an FDR cut-off of 0.05 is used for significance. On the other hand, DMRcate R-package (28), which is based on a kernel-smoothed estimator based on the limma results, was also used to identify DMRs between the three groups analyzed.

Finally, a functional analysis was performed using clusterProfiler R-package (29) by means of the over-representation analysis (ORA). We applied the analysis separately to the DMRs associated to genes and to promoters obtained from mCSEA, to the DMRs obtained by DMRcate in the septic shock vs. CIP analysis, for which all hypermethylated and hypomethylated regions were retrieved and separately enriched, and to the common DMRs identified by mCSEA and DMRcate in the septic shock vs. CIP comparison. The DMR sets were enriched in Gene Ontology (GO) terms and KEGG pathways, resulting in over-represented biological processes and metabolic pathways.

Heatmaps with hierarchical clustering of significant features were made with gplots and stats packages using heatmap.2 and hclust functions, respectively. PCAs were created with plotMDS function on limma R-package. Volcano plots are drawn with EnhancedVolcano R-package. All computational steps were performed using house-made R scripts.

### Validation of candidate gene methylation by bisulfite pyrosequencing

The DNA was obtained from patient leukocytes as previously described. This DNA underwent conversion treatment with bisulfite using the EpiTect Fast DNA Bisulfite Kit (Qiagen, Hilden, Germany). Next, 5μL of bisulfite-converted DNA was amplified by PCR, and subsequent pyrosequencing analysis was performed to analyze the methylation levels of each cytosine in the promoters of the selected genes, using the PyroMark PCR Kit (Qiagen, Hilden, Germany), for which both PCR amplification primers and pyrosequencing primers were designed (**Supplementary Table 1**). Subsequently, 10μl of the DNA amplified by PCR and 3μl of magnetic beads were loaded together. The pyrosequencing conditions were the standard ones provided by the equipment (Pyromark Q48 autoprep). The primer design was carried out using the PyroMark Assay Design 2.0 software. The parameters used for the design were as follows: primer size: 18–30 nucleotides; optimal primer size amplicon: 60-120 nucleotides; primer concentration: 0.2μM, hybridization temperature: 50-72°C, maximum hybridization difference between primers allowed: 10°C, maximum GC difference (%): 50. The design of the pyrosequencing analysis was carried out using the Pyromark Q48 Autoprep software.

### Statistical analysis

Statistics were calculated with SPSS v23 (IBM corporation), and Prism software (GraphPad Software Inc. San Diego, CA, USA).

Sample normality was determined with the Kolmogorov-Smirnov normality test. Since samples did not follow a Gaussian distribution, the Kruskal-Wallis test was used to analyze differences between the three groups (critically ill, sepsis, and septic shock patients), followed by a Dunns *post hoc* test, except for procalcitonin (PCT) values and lactate (6 hours) in which the non-parametric Mann Whitney U test was used. The Chi-square test was performed for ICU mortality.

## Results

### Clinical phenotypes of septic and septic shock patients

We used leukocytes isolated from blood samples of critically ill (CIP), septic, and septic shock patients, obtained prospectively by physicians from the ICU at *Hospital Clínico Universitario de Valencia* and stored in the INCLIVA Biobank. Clinical data from the different patient cohorts is summarized in **Table 1** (discovery cohort) and **Table 2** (validation cohort). CIP patients were diagnosed with intraparenquimatous hemorrhage (50% in the discovery cohort and 60% in the validation cohort) or subarachnoid hemorrhage (50% in the discovery cohort and 40% in the validation cohort). No other pathological conditions (i.e. chronic liver disease, chronic obstructive pulmonary disease, solid tumors, or immunosuppression) were found in the clinical records of this group of patients. Importantly, selected patients for this study were not treated with corticoids.

The patient groups showed similar demographic characteristics, comorbidities upon admission according to the Charlson Index, and disease severity. The APACHE II score did not show between-patient differences in the discovery cohort (**Table 1**), but statistical differences were found in the validation cohort (**Table 2**). In the discovery cohort, during the first day of ICU admission the median of the SOFA score was highest for septic shock patients with a value of 8.0 (5.5) compared to septic patients with 4.0 (6.0) and critically ill with 5 (2.5). The same results were obtained in the validation cohort (**Table 2**). We found the highest value for lactate at one hour in the septic shock group in both cohorts. (**Tables 1**, **2**). In the validation cohort we found that mortality was higher in septic shock patients (45.5%; 5 out of 11 patients) than in sepsis patients and in CIP (22%, 2 out of 9 patients). Particularly, in septic shock patients the group of non-survivors showed higher SOFA score, higher lactate levels and/or acute reactant levels such as PCT and CRP (**Supplementary Table 2**).

In the discovery cohort, between-group differences were found only in CRP values, and no differences were found for PCT between septic shock and septic patients (**Table 1**). In the validation cohort, clinical inflammatory parameter CRP was similar in the septic (median: 270.0, Interquartile range (IQR): 247.0) and septic shock groups (median: 235.0, IQR: 179.0), but was higher than in the CIP group (median: 4.8, IQR: 7.4). PCT levels were statistically higher in the septic shock group (median: 36.5, IQR: 59.0) than in the septic group (median: 2.1, IQR: 6.2). Both had a bacterial origin, although in the septic shock cases gut hypoperfusion and microorganism translocation could contribute to increases in this parameter (data not shown). The mortality rate in the ICU for the critically ill ranged from 22% in septic patients to over 45,5% in septic shock patients.

Microbiological documentation was possible in 100% of cases belonging to the discovery cohort and in the validation cohort it reached 70% of cases (**Supplementary Table 3**). Antibiotic therapy was administered within 1 to 6 hours of arrival at the emergency room and after the blood cultures were obtained.

Empirical therapy was prescribed based on the clinically suspected focus of infection. Basically, the antimicrobial therapies administered to sepsis and SS patients consisted in a combination of a betalactam plus a quinolone or a macrolide when community-acquired pneumonia was suspected being *Streptococcus pneumoniae* the main pathogen, and a broad spectrum betalactam plus an aminoglycoside or an oxazolidinone when abdominal, urinary, or unknown focus. In these cases, Enterobacteriaceae or polymicrobial infection use to be the etiology and broad spectrum with synergistic effect are preferred to get an appropriate treatment (detailed information of the antimicrobial therapy used is shown in **Supplementary Table 3**).

### Differential methylation in CpG sites in leukocytes from patient groups

**Figure 1:**
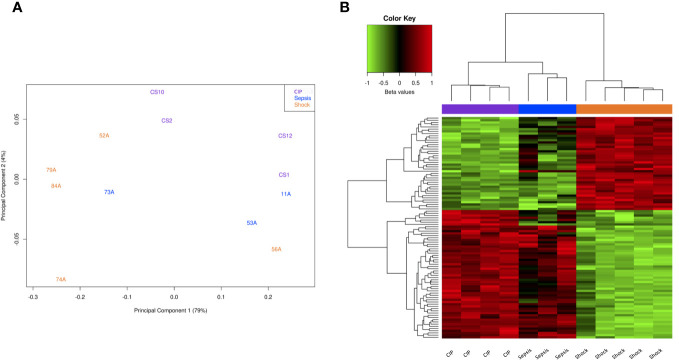
Methylation analysis at CpG level. **(A)** Principal Component Analysis (PCA) of the methylation data in critically ill, sepsis, and septic shock patients (purple, blue, and yellow, respectively). **(B)** Heatmap showing the methylation values (beta values scaled by the median) of the top 100 significant DMPs between septic shock and CIP. Red represents a higher methylation level, while green represents a lower methylation level.

First, to visualize the overall variation between samples, we generated a Principal Component Analysis (PCA) plot. **Figure 1A** illustrates the global differences in CpG methylation patterns among the three patient groups. As it can be seen, most septic shock patients formed a distinct cluster separated from critically ill and septic patients (**Figure 1A**). Septic patients also clustered separately from critically ill subjects, although sample 73A was distributed near to septic shock patients.

Second, we performed a differential methylation analysis at CpG level using limma. In the septic shock vs. CIP comparison, we identified 68,738 differentially methylated positions (DMPs) with an FDR < 0,05. Among these CpGs, 38,276 were found to be hypermethylated, while 30,462 displayed hypomethylation in the septic shock patients. In the comparison between sepsis patients and CIP, only 3 DMPs were found, whereas comparing septic shock and sepsis patients revealed 6 DMPs (see **Supplementary Material**).

Subsequently, we used the top 100 significant DMPs in the septic shock vs. CIP comparison to assess whether these differences were able to separate all the samples into their respective groups (**Figure 1B**). As can be seen, the samples were grouped into 3 clusters that correspond to their phenotypic groups.

**Figure 2:**
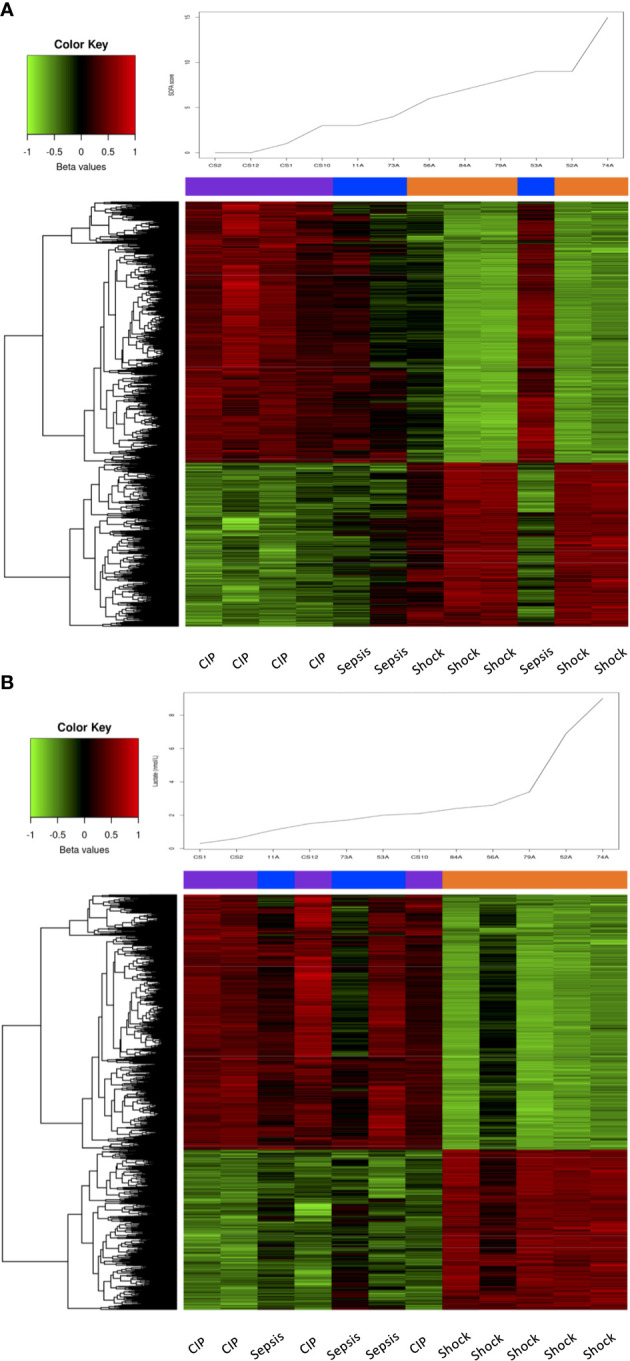
Association of SOFA and lactate levels with DNA methylation changes. **(A)** Heatmap showing the methylation values (beta values scaled by the median) of the 6657 DMPs with an FDR < 0.01 in septic shock vs. CIP comparison, sorted by the samples’ SOFA score, which is plotted at the top of the heatmap. **(B)** Heatmap showing the methylation values (beta values scaled by the median) of the 6657 DMPs with an FDR < 0.01 in septic shock vs. CIP comparison, sorted by the samples’ lactate level, which is plotted at the top of the heatmap.

An interesting association was found while analyzing the DNA methylation profiles along with the SOFA score (a widely used measure for assessing organ dysfunction in sepsis management). The DNA methylation patterns of the 6,657 DMPs with an FDR < 0,01 found in the Septic shock vs. CIP comparison were able to effectively classify the different patient groups based on their SOFA score, except for one septic patient (53A) who showed a high SOFA score value of 7 (**Figure 2A**). We further conducted analysis to examine the association between DNA methylation patterns and lactate levels within the first hour, given that lactate levels over 2 mmol/L serve as an effective parameter for classifying septic shock patients (1). The results demonstrated a strong association between DNA methylation changes and lactate levels. Notably, the 6,657 DMPs accurately classified septic shock patients according to their lactate levels, indicating a highly specific relationship. However, it was not possible to distinguish septic from critically ill patients based on DNA methylation signature, as the two groups exhibited similar methylation patterns (**Figure 2B**). This observation aligns with the fact that lactate levels tended to be in the same range in septic and critically ill patients.

Overall, these results suggest a link between substantial alterations in DNA methylation patterns and disease severity according to total SOFA score and lactate levels.

### Substantial methylation changes at DMRs in septic shock patients

To identify DMRs within our patient cohorts, we first employed mCSEA analysis (27), which revealed a total of 1,619 DMRs (801 promoters and 926 genes) in septic shock patients compared to CIP, 245 DMRs (102 promoters and 148 genes) in sepsis patients compared to CIP, and 1,464 DMRs (821 promoters and 732 genes) in septic shock vs. sepsis patients. These findings indicate that the most substantial changes to DNA methylation profile are experienced in patients with septic shock. Additionally, DMRcate analysis identified a total of 8,373 DMRs, encompassing genes and promoters, in the comparison between septic shock and CIP, surpassing the number of DMRs obtained by mCSEA in this comparison. Among these regions, hypomethylation was found in 4,378 septic shock samples, while 3,995 showed hypermethylation. Notably, there were 609 regions with a mean beta value difference of more than 15%. No significant DMRs were found in the sepsis vs. CIP comparison, and only 1 DMR was found in the septic shock vs. sepsis comparison.

**Figure 3:**
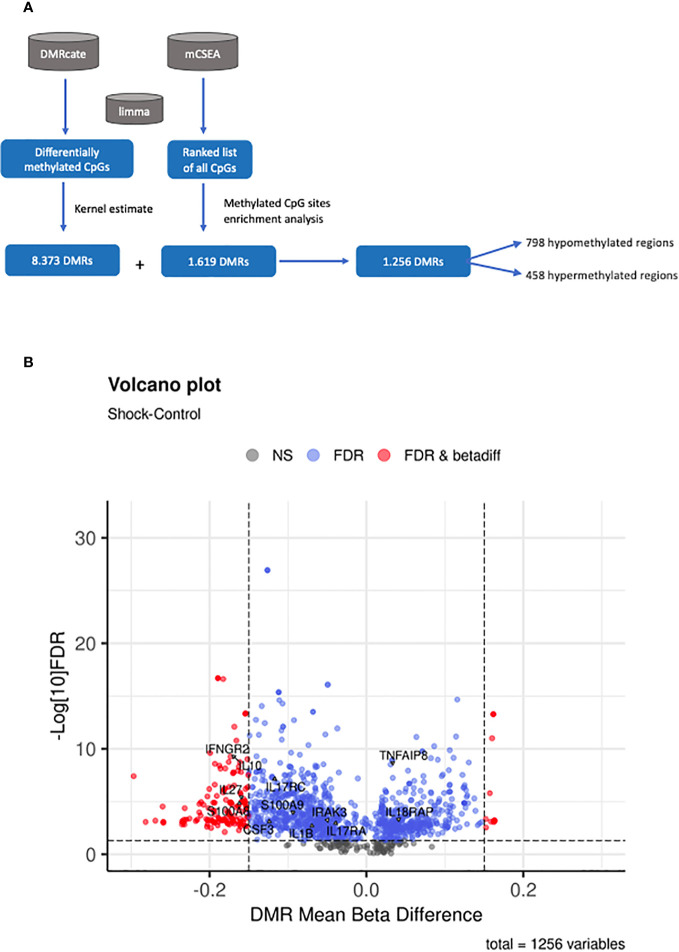
Methylation analysis at region level. **(A)** Summary of the workflow followed for the identification of DMRs. **(B)** Volcano plot of the 1256 DMRs obtained by both DMRcate and mCSEA approaches, showing the mean beta-value difference between shock septic and CIP in the x-axis and the -log10 FDR in the y-axis. FDR and beta-values are retrieved from the DMRcate output. Points in black correspond to non-significant regions; points in blue are significant DMRs, and those in red are significant DMRs with a mean beta-value difference higher than 0.15 between shock septic and CIP.

The comparison of the two DMRs sets generated using mCSEA and DMRcate revealed a total of 1,256 matching regions between the two analyses. Among these regions, 798 were found to be hypomethylated, while 458 were hypermethylated (**Figure 3A**). **Figure 3B** shows a volcano plot representing the differential methylation results for the 1,256 DMRs common to both approximations.

### Functional and biological implications of DNA methylation changes occurring in sepsis and septic shock patients

To gain insight into the biological implications of the DMRs, we performed an enrichment analysis to identify the key biological processes underlying sepsis and septic shock. To assess the robustness of the results, we analyzed the DMRs obtained by mCSEA, the DMRs obtained by DMRcate, and the common DMRs obtained by both approximations.

First, enrichment analysis of the DMRs obtained by mCSEA revealed KEGG molecular pathways and biological processes related to several pathological conditions which were associated with inflammatory response and the immune system (**Supplementary Figure 1**). Notably, we observed significant enrichment in KEGG pathways related to cytokine-cytokine receptor interaction in septic shock compared to critically ill patients. In the case of sepsis vs. CIP, KEGG pathways such as T cell receptor signaling pathway and Th17 cell differentiation were of particular interest (**Supplementary Figures 1A–C**). Additionally, the study of GO biological processes (**Supplementary Figures 2A–C**) uncovered a range of pathways involving both genes and promoters. These pathways included T-cell activation, lymphocyte differentiation, neutrophil activation and degranulation, positive regulation of cytokine production, and regulation of the inflammatory response. These findings were consistent across all three patient groups studied.

Second, we also performed an enrichment analysis on the DMRs obtained from the septic shock vs. CIP comparison using DMRcate. This analysis revealed significant GO categories such as neutrophil-mediated immunity, T cell activation, leukocyte cell-cell adhesion, response to molecule of bacterial origin, regulation of cysteine-type endopeptidase activity involved in the apoptotic process, regulation of innate immune response, and positive regulation of NF-kappaB transcription factor activity. Enrichment of these DMRs also resulted in various KEGG pathways, including T cell receptor signaling pathway, PD-L1 expression and PD-1 checkpoint pathway, C-type lectin receptor signaling pathway, TNF signaling pathway, natural killer cell-mediated cytotoxicity, NF-kappa B signaling pathway, Th1 and Th2 cell differentiation, Th17 cell differentiation, hematopoietic cell lineage, and cytokine-cytokine receptor interaction.

**Figure 4:**
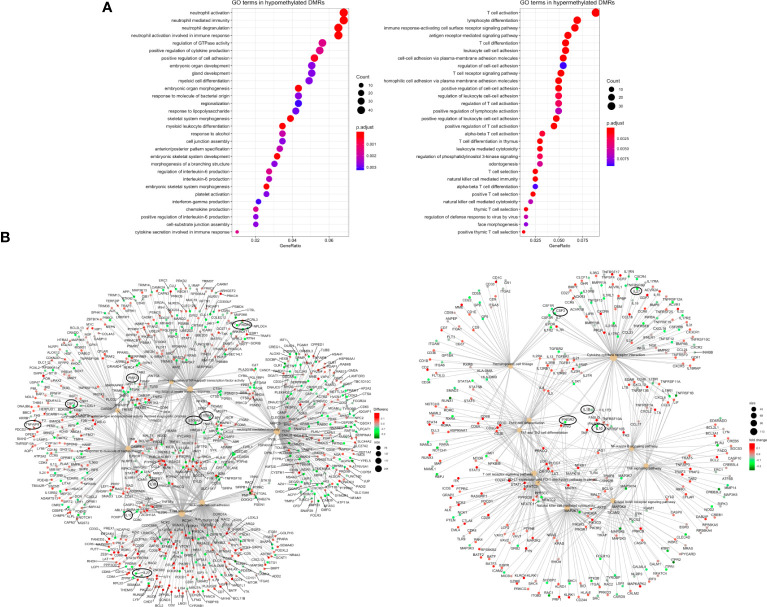
Pathway enrichment analysis **(A)** Dotplot with the most significant Gene Ontology-Biological Process (GO-BP) terms obtained by ORA for the 1256 common DMRs between septic shock and CIP, separated in hypomethylated (left) and hypermethylated (right) DMRs. The y axis represents the GO-BP terms, and the gene ratio in the x axis represents the proportion of genes involved in each biological process over the total number of DMRs obtained (hypermethylated and hypomethylated, respectively). The size of each dot or gene cluster depends on the gene count contributing to the enrichment of that term. The adjusted p-value obtained from enrichment analysis for each term in Fisher’s exact test determines the color of the dots. **(B)** Gene-Concept Networks (cnetplots) of ORA from DMRs obtained by DMRcate. The cnetplots include 7 enriched GO-BP terms (left) and 10 enriched KEGG pathways (right) related with sepsis and their linkage to the significant DMRs (from septic shock vs. CIP comparison) involved in each GO-BP term or KEGG pathway. The size of the nodes depends on the gene count involved in the GO-BP term or KEGG pathway; while the genes are colored according to whether they are hyper (red) or hypomethylated (green).

Finally, analysis of the top 10 GO terms associated with the common 798 hypomethylated regions revealed important biological processes, including neutrophil-mediated immunity, positive regulation of cytokine production, positive regulation of cell adhesion, regulation of IL-6 production, regulation of TNF (tumor necrosis factor) production, interferon-gamma production, response to molecule from bacterial origin, and regulation of hematopoiesis. The 458 common hypermethylated regions were enriched in GO terms such as T cell activation, lymphocyte differentiation, immune response through cell surface receptor signaling pathway, cell-to-cell adhesion via plasma-membrane adhesion molecules (i.e., protocadherins), antigen-receptor-mediated signaling pathway, and natural killer cell-mediated immunity (**Figure 4A**).

As a further step, the results were visualized in gene-concept networks (**Figure 4B**), which depicted the connections between GO terms and the DNA methylation changes observed in specific genes involved in the immune system. Among the individual genes exhibiting significant differential methylation, *IL10*, *IL27*, *IL1B*, *IRAK3*, *CSF3*, *IFNGR2*, *IL17*, *S100A8* and *S100A9* were found to be hypomethylated, while *TNFAIP8* and *IL18RAP* were hypermethylated (**Figures 3B**, **4B**). These genes are linked to key biological processes involved in the immune response, which can lead to dysregulated immune responses.

Given that some of these genes were also reported by Severino et al. (30) and Lu et al. (30, 31), we subsequently investigated DMRs potentially regulating the expression of these genes, focusing our attention especially on promoters and relevant regulatory regions.

Our results revealed the existence of DMRs in the promoters of pro-inflammatory cytokines (such as *IL1B*, *IL18*, *TNFAIP8*, and *IL17A*), anti-inflammatory cytokines (such as *IL10*), and immunosuppression mediators, including members of the IL10 superfamily (*S100A8* and *S100A9*) (**Figures 3B**, **4B**). Notably, changes in DNA methylation were consistently observed across all CpG sites within the promoter regions of the analyzed genes. These candidates were selected for DNA methylation validation in an independent cohort of subjects based on their statistical significance (FDR <0.05). These selected genes were predominantly involved in inflammatory response and immunosuppression events, reflecting their potential significance in sepsis pathophysiology.

### Altered DNA methylated patterns involved in critical immune system-related pathways in septic shock patients

To explore the most relevant genes with differential DNA methylation among the three groups, we conducted pyrosequencing experiments to corroborate the methylation patterns that regulate the promoters of these key genes displaying the highest degree of differential methylation.

As described in the previous section, we selected genes showing differential methylation in between-group comparisons, appearing in enrichment analysis in the cnetplots maps and with relevant roles in immunosuppression. Genes selected from the previous section were investigated in terms of their biological relevance in sepsis, based on the different biological processes they were involved in, and the previous findings we and other authors have reported (3–5). This strategy allowed us to select the 10 most relevant candidates (*ILB1, IL10, IL17, IL18, IFNGR2, S100A8, S100A9, TNFAIP8, TREM1, IRAK3*) for further validation through pyrosequencing. Among these selected candidates, *S100A9* and *IRAK3* showed no amplification in the analyzed promoter regions. Note that *S100A9* functions as a dimer together with its isoform *S100A8*, and *IRAK3* codifies for a receptor of IL-1, whose family members IL-1β and IL18 were also studied, enabling us to infer some of the altered functions of these mechanisms.

**Figure 5:**
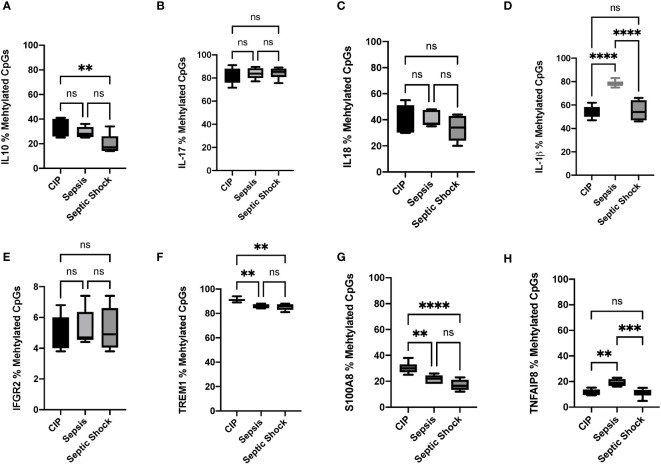
Percentage (%) of methylated CpGs of different pro-inflammatory, anti-inflammatory, and immunomodulatory cytokines in leukocytes obtained from critically ill, sepsis, and septic shock patients. **(A)** Gene promoter methylation levels of IL-10 CpGs, **(B)** IL17, **(C)** IL18, **(D)** IL1β, **(E)** IFGR2, **(F)** TREM1 **(G)** S100A8, and **(H)** TNFAIP8. *P < 0.05; **p < 0.01, ***p < 0.001, ****p < 0.00001. ns, not significant.

The results obtained in pyrosequencing experiments confirmed the hypomethylation status observed in the bioinformatics analysis for most of the analyzed methylated regions, thereby validating our previous findings. Specifically, we observed statistically significant differences in the methylation status of *IL10* between the CIP and septic shock groups, with the septic shock group showing around 20% hypomethylation compared to non-septic critically ill patients (**Figure 5A**). Similarly, the septic shock group exhibited hypomethylation in the *S100A8* gene (15%) and the *TREM1* gene (7%) compared to the CIP group (**Figures 5G, F**, respectively). In the comparison between septic shock and sepsis groups, we found hypomethylation of *IL1B* (septic shock group 20% hypomethylated compared to sepsis group) (**Figure 5D**) and *TNFAIP8* (septic shock group 10% hypomethylated compared to sepsis group) (**Figure 5H**). When comparing sepsis and CIP groups, sepsis exhibited hypermethylation in the *TNFAIP8* gene (approximately 10% hypermethylated in the septic group), hypomethylation in the *IL1B* gene (20% hypomethylated in the sepsis group), and hypomethylation in the *TREM1* gene (7% hypomethylated in the sepsis group) (**Figures 5H, F**, respectively). No differences were observed between septic shock and CIP groups in the promoter regions of the immunomodulatory genes *IL1B*, *TNFAIP8*, *IL17*, *IL18*, and *IFGR2* (**Figures 5D, H, B, C, E**, respectively). Similarly, no differences were found in the promoter regions of the *IL10*, *IL17*, *IL18*, *IFGR2*, and *S100A8* genes in the sepsis group compared to CIP (**Figures 5A–C, E, G**).

## Discussion

Sepsis is a complex and heterogeneous clinical entity which involves organ dysfunction due to a dysregulated and exacerbated immune response to infection (32). This is sometimes followed by host immunosuppression, which increases the risk of death and leads to several comorbidities in survivors (33). Exploring DNA methylation patterns in immunity cells may help elucidate the relationship between epigenetics and immune responses, providing insight into the intricate mechanisms involved in immunosuppression.

The immunosuppression promoted by a septic episode (34) is the consequence of the simultaneous hyper-inflammatory response inducing failure or exhaustion of the innate and adaptive immune system (35). Immunosuppression therefore helps compensate for the excessive production of pro-inflammatory cytokines and immunomodulatory chemokines in hosts, and may last for months or even years (6, 36).

Different authors have reported several mechanisms involved in immunosuppression, such as changes in the glycolytic metabolism of leukocytes (37), increased apoptosis in immune cells such as macrophages and lymphocytes (38), alterations in myeloid cell differentiation, increased Treg cell count (39, 40) and MDSC proliferation (41), some of which have also been detected in our study. However, although several mechanisms have been associated with immunosuppression, critical information remains unknown. Future research efforts should focus on further characterizing immunosuppression mechanisms and developing diagnostic tools and therapies for the long-term survival of immunosuppressed patients. Consequently, mechanisms that can modify gene expression patterns are of particular interest for understanding the immunosuppressive phenotype originated by sepsis. In this regard, epigenetics has emerged as a potential tool to identify new mechanisms and molecular pathways altered during sepsis (17), and to develop new diagnostic and prognostic tools to identify immunosuppressed patients who could benefit from a specific therapeutic approach.

In this study, we found specific changes in leukocyte DNA methylation patterns in different patient groups, mainly septic shock patients. It is noteworthy that one limitation of our study is the low number of samples used in the discovery cohort, which may impact the number of DNA methylation changes observed in our comparisons. In particular, the fact that two of the three sepsis patients grouped with CIP, while the other one grouped with septic shock patients, may explain the low number of significant DMPs obtained in the comparisons that involve the sepsis group. The most substantial DNA methylation changes were seen in genes associated with the immune response and inflammatory signaling pathways, as well as different critical immune cell receptors and pathways involved in T helper cell regulation. Some of these genes have been previously described as upregulated by Severino et al. (30) and more recently by Lu et al. (31), using differential gene expression data from patients with gram-negative sepsis vs. healthy individuals [accession n°: GSE69528] (42) [accession n° GSE46955] (43). In this regard, our results agree with previous studies by Severino et al. and Lu et al., in which genes such as *S100A8* or *S100A9* were hypomethylated in sepsis and septic shock and were consequently upregulated in these conditions (30, 31). In the present study, we found altered global DNA methylation patterns closely related with organ disfunction and lactate levels among the different patient groups, demonstrating a direct relationship between the DNA methylation patterns and clinical phenotype severity. These results are in consonance with our previous work (22), in which we showed a direct relationship between DNA methylome alterations and clinical severity in neonatal sepsis. Importantly, there is evidence that lactate can alter several epigenetic mechanisms such as chromatin conformation (i.e., histone lactylation) (44) and TET (Ten Eleven Translocation) activation, leading to hydroxymethylation and subsequent hypomethylation of the DNA (44, 45).

The dynamics of DNA methylation patterns in patients with septic shock present a complex and multifaceted picture. While DNA methylation changes can indeed occur rapidly in response to various stimuli, including immune processes like inflammation, it is plausible that pre-existing methylation patterns play a pivotal role in the progression of sepsis to septic shock. The rate and magnitude of these methylation alterations are subject to a multitude of factors, including the specific genes involved, the nature and intensity of the immune response, and the targeted cell types. Notably, during the evolution of sepsis to septic shock, methylation patterns become dysregulated, likely due to the heightened inflammatory response and cellular stress intrinsic to the disease (17). Such dysregulation can potentially contribute not only to the development and severity of septic shock but also to long-term immunosuppression, since while some DNA methylation changes linked to the inflammation and immune response are transient and reversible, others have enduring consequences. These persistent alterations establish an ‘immunological memory,’ thereby influencing future immune responses. Furthermore, within an inflammatory milieu, immune cells like macrophages and T cells can release cytokines that profoundly impact the DNA methylation patterns of nearby cells (46), modulating the expression of specific genes involved in the immune response and therefore contributing to the severity of disease.

As regards the functional and biological implications of the DNA methylation changes observed in septic patients (especially in the septic shock group), these alterations were found to be related to T cell hyper-responsiveness. This is a key finding, since continuous T cell activation has been linked to exhaustion of the adaptive immune system and therefore an abrupt drop in ability to respond to several stimuli, leading ultimately to immunosuppression (47). In addition, we found hypermethylation in some genes coding for proteins like PD-1 and PD-L1, which have been historically associated with an exhausted immune system (48–50). Focusing on T cells, we found that the T cell group most affected by changes in DNA methylome are T helper cells. These cells are critical players in adaptive immunity, being required for almost all adaptive immune responses. In fact, T helper cells play an important role in activating B cells to secrete antibodies, as well as helping macrophages to destroy microbes and even mediating cytotoxic T cell activation to kill infected target cells (51). Alterations in the normal function of T helper cells are likely to cause an immunosuppression phenotype. As well as changes in the DNA methylome of T cells, we found that other immune cells such as macrophages also experience remodeling in their ability to respond to different stimuli, and consequently some functions are altered. As is well known, during primary responses to bacteria macrophages produce pro-inflammatory IL-1β, IL-18 and TNF-α for microorganism clearance. Following lipopolysaccharide (LPS) tolerance induction, it has been demonstrated that chromatin in macrophages is enriched in repressive histone post-translational modifications (i.e., H3K9me2) at the promoter regions of *IL1B* and *TNFA* (52, 53). Our results indicate that during the early stages of septic shock (first 24 h after ICU admission, when the blood samples were obtained) the *IL1B* promoter was hypo-methylated in septic shock patients compared to sepsis patients, so suggesting *IL1B* would be highly expressed in septic shock patients, as we recently described (3). IL-1β is another cytokine that mediates pro-inflammatory states but also plays an immunomodulatory role, so the hypomethylation of its gene may increase protein expression in septic shock patients, as we have previously demonstrated (3). Regarding *TNFA*, we found hypomethylation at the promoter region in the septic shock group compared to septic group, indicating that the methylation status may be associated with the increased circulating TNFα levels found in septic shock patients compared to septic patients, as we also described in patients admitted in the ICU at the first hours of ICU admission (3). However, simultaneous to the overactivated pro-inflammatory cytokines found in the sepsis groups, we also report DNA methylation changes involving the anti-inflammatory cytokines. The increase of anti-inflammatory cytokines (such as TGF-β and IL-10) and T_H_ 1-opposing T_H_ 2 cytokines (such as IL-13) during CARS is useful to counteract the proinflammatory responses of SIRS. Moreover, cytokine IL-10 inhibits the expression of Class II major histocompatibility complex (MHC) proteins (HLA-DR, HLA-DMA, and HLA-DMB) on the surface of macrophages and monocytes, which in addition to TGF-β, suppresses T-cell proliferation, contributing to immunosuppression in sepsis. In this regard, it has been demonstrated that HLA-DR expression is suppressed in most patients with sepsis at early stages, but recovers within ten days in survivors (54). We found the hypermethylation of specific regions in the gene body of a family of HLA (i.e., HLA-E, HLA-DMB, HLA-DQB2) and also in the promoter of HLA-DMB in septic shock patients at the time of ICU admission. HLA are heterodimeric molecules which are important for normal antigen presentation. Although we have not measured the expression levels of HLA genes, hypermethylation of these elements may produce the downregulation of HLA expression, and contribute to immunosuppression events, as previously observed by Tschaikowsky et al. (54) and more recently has been proposed by Yao et al., who described how monocytes with low expression of HLA-DR and S100A correlated with immunosuppressive state upon septic challenge (55). Other authors such as Hiraki et al. established that IL‐10 levels positively correlate with Treg cell percentages in the CD4+ T‐cell population in patients with postoperative infections (56). Based on our results, *IL10* gene hypomethylation also correlated with elevated IL-10 levels in plasma of septic patients, as we recently demonstrated (3), supporting the idea that alterations in methylation levels induce changes in the immune system response during sepsis progression. In addition, IL-10 levels are important because of the role of the anti-inflammatory IL-10 by inhibiting the expression of pro-inflammatory mediators such as TNF-α and IL-1β, and by conferring diminished resistance to pathogenic organisms during sepsis (57).

Another key factor contributing to immunosuppression is MDSC proliferation, which induces strong immunosuppressive states in several diseases such as cancer, inflammation, and infection diseases including sepsis (41, 58, 59). In this regard, in our study we found genes involved in MDSC suppressive functions, such as soluble factors *S100A12*, *S100A8*, *S100A9*, *MMP8*, granulocyte colony–stimulating factor (*GCSF*), *IL6*, *IL10*, vascular endothelial growth factor (*VGEF*), *TFGB* and *ARG1*. Interestingly, some of these factors were also found to be up-regulated in the peripheral blood of septic patients in our and other previous studies (3, 16). Our finding that *S100A8*, *S100A9*, and *IL10* promoters were hypomethylated in EPIC850K arrays at early stages of sepsis and septic shock (*IL10* and *S100A8* hypomethylation were also validated by pyrosequencing in the independent cohort) therefore suggested that these immunosuppression events occur concomitantly with the early cytokine storm. We also found hypomethylation in the *CSF3* gene, granulocyte colony-stimulating factor 3 and G-CSF, the major regulator of neutrophil production under basal conditions of hematopoiesis and also essential for granulopoiesis in response to bacterial infections to enhance multiple neutrophil functions and prevent overwhelming neutrophil invasion during infection (60). T cells induce G-CSF production through IL-17 release. Our results show an altered methylation status of *CSF3* and *IL17* in EPIC850K arrays, although these results could not be validated using pyrosequencing. Here it is important to underline the possibility that the primers designed for pyrosequencing assays were not able to measure the same CpGs evaluated in the array. In any case, IL-17 indubitably plays a critical role as a bridge between innate and adaptive immune systems, therefore contributing to long-term immunosuppression (61).

Another relevant gene found in the methylation array with altered DNA methylation was *IRAK3*, the IL-1 receptor-associated kinase M. *IRAK3* is an immune-associated protein which negatively regulates Toll-like receptor signaling and mediates critical aspects of innate immunity, resulting in an immunocompromised state during sepsis (62). Importantly, *IRAK3* expression is elevated in blood monocytes from patients with sepsis (31, 63), and even more so in septic shock (64). Our results showed hypomethylation of the *IRAK3* promoter in sepsis and septic shock, which could contribute to increased circulating *IRAK3* in septic and septic shock patients, thus explaining the elevated levels found in these patients.

The present study provides insight into the way DNA methylation can control the expression of key genes participating in molecular pathways which guide immune response and immunosuppression during the first stages of the septic process, especially in cases of critically ill patients with septic shock.

## Conclusions

The differential methylation analysis at position level enabled us to clearly distinguish sepsis and septic shock from critically ill patients with the top 100 DMPs obtained. Moreover, we found a strong association of organ dysfunction and lactate levels with these global DNA methylation changes in leukocytes. The differential methylation analysis at region level used two different approaches (DMRcate and mCSEA) to identify DMRs, revealing a total of 1,256 genes, related to the inflammatory and anti-inflammatory response, immune cell differentiation and activation and immunosuppression, with a differential DNA methylation status between groups. Finally, we were able to validate this methylation differences by bisulfite pyrosequencing in *IL10*, *IL1B*, *S100A8* and *TNFAIP8*. Among these, *IL10* and *S100A8*, which are closely related with immunosuppression, were hypomethylated in septic shock patients.

## Data availability statement

The datasets presented in this study can be found in online repositories. The names of the repository/repositories and accession number(s) can be found below: GSE247524 (GEO).

## Ethics statement

The Committee for Human Subjects of the Hospital Clínico Universitario de Valencia (Valencia, Spain) approved the study (reference number 2019/051). The studies were conducted in accordance with the local legislation and institutional requirements. The participants provided their written informed consent to participate in this study.

## Author contributions

JB-G: Data curation, Formal analysis, Investigation, Validation, Writing – original draft. PN-L: Formal analysis, Investigation, Writing – original draft. RO-V: Formal analysis, Investigation, Writing – original draft. GC-V: Formal analysis, Investigation, Methodology, Writing – original draft. CF-S: Data curation, Formal analysis, Writing – original draft. MR-G: Data curation, Formal analysis, Writing – original draft. EN-S: Formal analysis, Investigation, Writing – original draft. EG-L: Data curation, Formal analysis, Writing – original draft. FP: Writing – review & editing. NC: Conceptualization, Formal analysis, Validation, Writing – review & editing. SM-M: Conceptualization, Formal analysis, Investigation, Writing – review & editing. JG-G: Conceptualization, Funding acquisition, Project administration, Writing – original draft, Writing – review & editing.
