# Supplementary material for: Alterations in leukocyte DNA methylome are associated to immunosuppression in severe clinical phenotypes of septic patients
Source: Front Immunol. 2024 Jan 3;14:1333705. doi: 10.3389/fimmu.2023.1333705 (PMC10791922; doi:10.3389/fimmu.2023.1333705)
Supplement: Supplementary file 1 [file DataSheet_1.pdf]

Supplementary Material

Supplementary Table 1. Set of primers used in the pyrosequencing experiments. F means "Forward", R means "Reverse" and S means "Sequencing".

Gane	Secuence
Il-1b_F	GTTTGGTATGTATATTTAGGTGGTAGAGT
IL-1b_R	ACTCTTTTTCCCACAATAAAACCTATTT
IL-1b_S	TTTATTTAAATTAGTATGTGTTAGG
IL-10_F	GTTGATTTTTTGGGGAGAATAGTT
IL-10_R	AAAACCCAATTATTTCTCAATCCCATTAT
IL-10_S	GGGGAGAATAGTTGTT
IL-17_F	GTGAGATGTTTTTGTTGATTTGGATTAGAT
IL-17_R	ACCAAAACTTTCATTTCCTATCC
IL-17_S	TGTTGATTTGGATTAGATAGTA
IL-18_F	GGTATAGGTTTTGGAAGGTATAGAG
IL-18_R	CTTAAACCCCCCTTCAAACAAACTCTT
IL-18_S	TGGAAGGTATAGAGTTTTAA
INFGR2_F	GGGGGAGAGTTGAAAGTTTAA
INFGR2_R	TAAAACCCCCACCCCTCCTAA
INFGR2_S	TGGGGGTTATTAAAGTAAA
S100A8_F	TTAGGTGAATGTGGTAATTATTGTGTAGA
S100A8_R	AAAACCCCCCTACCTACTTTTTC
S100A8_S	AATTTTGGGTGGGGA
S100A9_F	TGAATTAGGGGGAATTTAAAGAGT
S100A9_R	CCCATCCTCCCTTATCAAC
S100A9_S	CCTTATCAACCCAAACT
TNFAIP8_F	AGAAGAGGATGGGAGTTTTAAAG
TNFAIP8_R	TTTTCCTCCCCTCCCCTCTAA
TNFAIP8_S	ATGGGAGTTTTAAAGTGA
TREM1_F	GGGAGAATATGTAGTGTTTGTATATAAAAG
TREM1_R	ACTCTATCATTATTCCCATACTAAACTTT
TREM1_S	AGTTAGGGAAAAAAGATG

Supplementary Table 2. Clinical data from septic shock patients in the validation cohort

Validation cohort			
Participant characteristics	Septic shock-survivors	Septic shock Non-survivors	p-value
Cases (%)	6 (55%)	5 (45%)	
SOFA score 1st day Median (IQR)	7.5 (1.75)	14 (3)	0.007686
Lactate 1st hour (mmol/l) median (IQR)	3.05 (0.175)	6 (0.7)	0.007969
Procalcitonin (ng/mL) median (IQR)	22.95 (16.075)	50.8 (10)	0.004329
CRP (mg/l) median (IQR)	180 (13.25)	221 (22)	0.0303

Note: IQR: interquartile range. P-values are obtained from Mann–Whitney–Wilcoxon non-parametric test

Supplementary Table 3. Microorganisms and antimicrobial therapy administered to patients" in the discovery Cohort and the validation cohort.

**Discovery cohort**			
**Number of patient/ Description**	**Microorganism**	**Antibiotic 1**	**Antibiotic 2**
11 Sepsis	*E. coli*	meropenem	
73 Sepsis	*S. aureus*	ceftriaxone	levofloxacin
53 Sepsis	*S. pneumoniae*	ceftriaxone	levofloxacin
52 SS	*S. pneumoniae*	ceftriaxone	azithromycin
56 SS	*E. coli*	meropenem	amikacin
74 SS	*S. pneumoniae*	ceftriaxone	clindamycin
79 SS	*E. coli*	ceftriaxone	amikacin
84 SS	*E. coli*	piperacillin/tazobactam	amikacin
**Validation cohort**			
4 Sepsis	No filiated	ceftriaxone	levofloxacin
11 Sepsis	*E. coli*	meropenem	
18 Sepsis	No filiated	amoxicillin/clavulanic	levofloxacin
29 Sepsis	Influenza A	ertapenem	levofloxacin
34 Sepsis	No filiated	ceftriaxone	azithromycin
35 Sepsis	No filiated	meropenem	amikacin
44 Sepsis	*S. bovis*	ceftriaxone	levofloxacin
45 Sepsis	*S. pneumoniae*	ceftriaxone	levofloxacin
50 Sepsis	No filiated	ceftriaxone	azithromycin
2 SS	*E. coli*	meropenem	amikacin
10 SS	No filiated	ceftriaxone	levofloxacin
15 SS	*K. pneumoniae*	meropenem	amikacini
17 SS	*S. aureus*	ceftriaxone	piperacillin/tazobactam

19	SS	*S. pneumoniae*	ceftriaxone	azithromycin
22	SS	*S. mitis*	meropenem	metronidazole
25	SS	No filiated	meropenem	linezolid
26	SS	No filiated	meropenem	linezolid
30	SS	*S. pneumoniae*	piperacillin/tazobactam	levofloxacin
33	SS	*K. pneumoniae*	meropenem	amikacin
38	SS	*K. oxytoca*	meropenem	levofloxacin

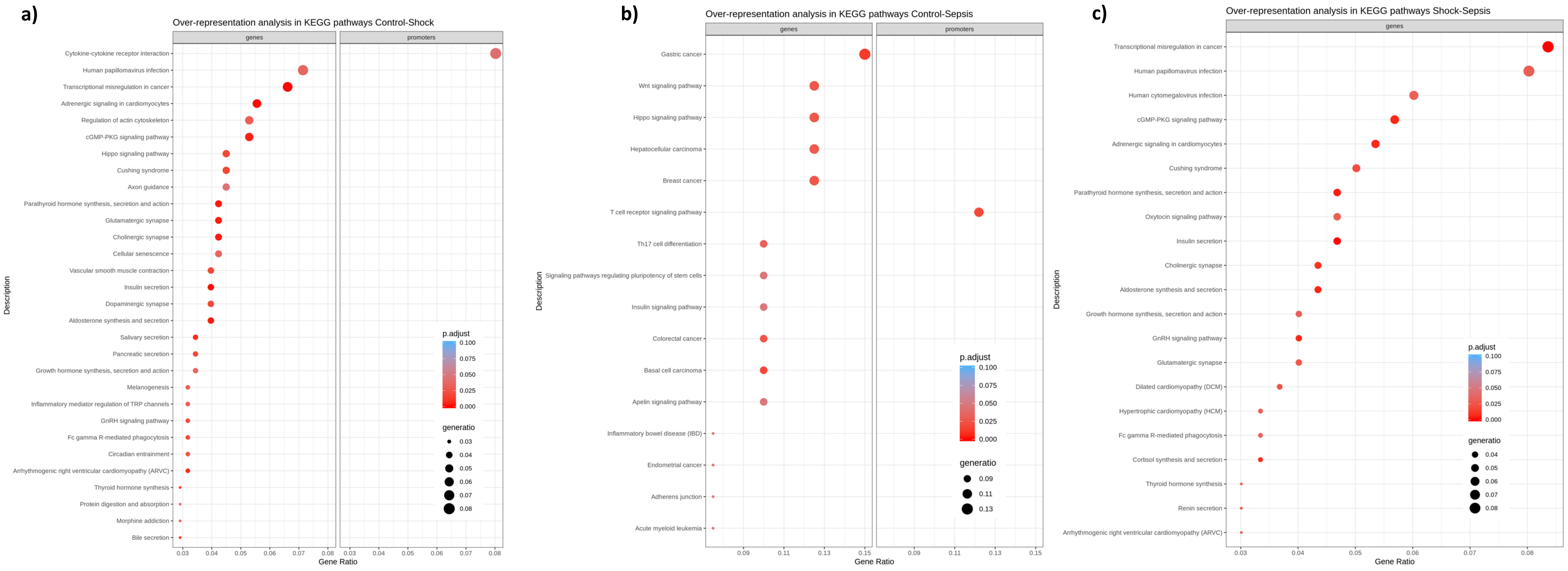

Supplementary Figure 1. Over-representation analysis using mCSEA approach. A). Dotplots from significant KEGG pathways obtained by an over-representation analysis for the DMRs, divided between genes and promoters for the three comparisons: a) Septic shock vs. CIP b) Sepsis vs. CIP; and c) septic shock vs. sepsis. The y axis represents the top 10 enrichment results, and the gene ratio in the x axis represents the proportion of regions involved in the metabolic pathway over the total number of DMRs. The size of each dot or gene cluster depends on the count of genes that contribute to the enrichment of that pathway. The adjusted p-value obtained for the terms in the Fisher's exact test of the over-representation analysis determines the color of the dots.

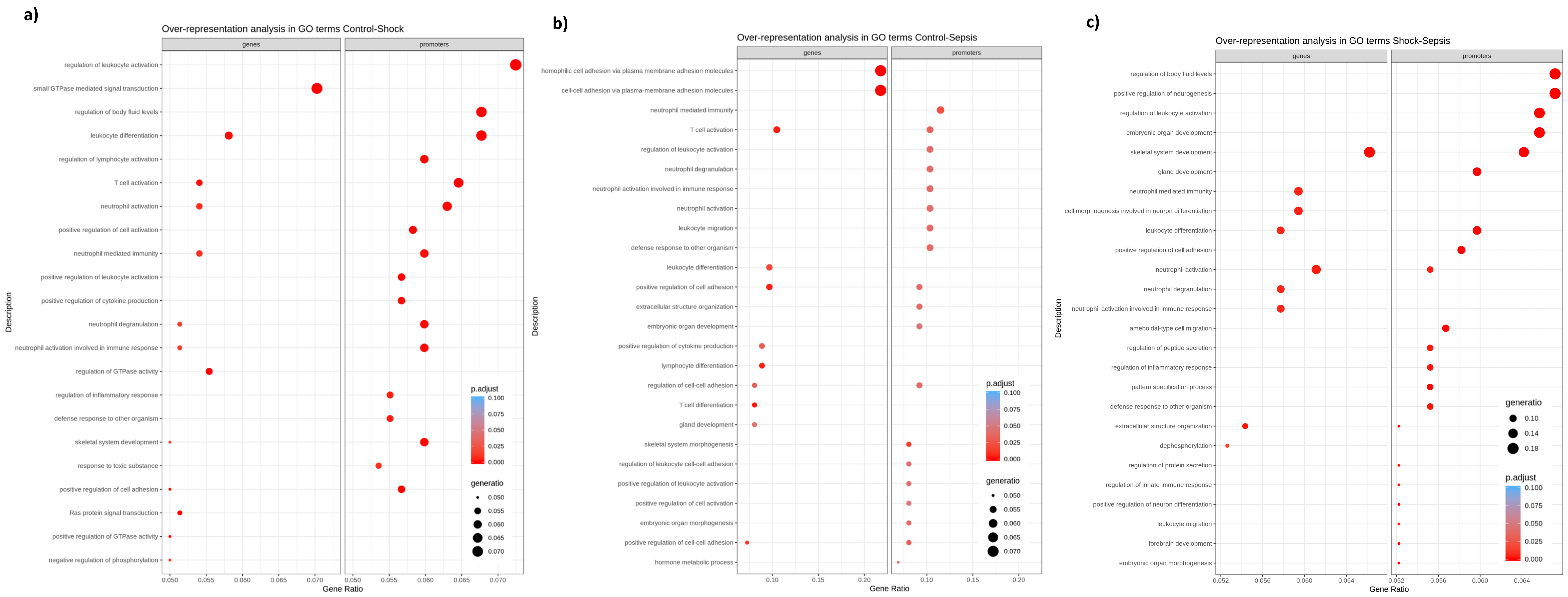

Supplementary Figure 2. Over-representation analysis using mCSEA approach. Dotplots of the top 20 significant GO-BP (biological process) terms obtained for the DMRs (genes and promoters) in the three comparisons: a) Septic shock vs. CIP b) Sepsis vs. CIP; and c) septic shock vs. sepsis. The y axis represents the top 10 enrichment results, and the gene ratio in the x axis represents the proportion of regions involved in the metabolic pathway over the total number of DMRs. The size of each dot or gene cluster depends on the count of genes that contribute to the enrichment of that pathway. The adjusted p-value obtained for the terms in the Fisher's exact test of the over-representation analysis determines the color of the dots.
